# Groundwater Arsenic Distribution in India by Machine Learning Geospatial Modeling

**DOI:** 10.3390/ijerph17197119

**Published:** 2020-09-28

**Authors:** Joel Podgorski, Ruohan Wu, Biswajit Chakravorty, David A. Polya

**Affiliations:** 1 Department of Earth and Environmental Sciences and Williamson Research Centre for Molecular Environmental Science, University of Manchester, Manchester M13 9PL, UK; ruohan.wu@postgrad.manchester.ac.uk; 2 Centre for Flood Management Studies, National Institute of Hydrology, Water and Land Management Institute Complex, Phulwarisharif, Patna, Bihar 801505, India; biswajitnh@gmail.com

**Keywords:** groundwater, arsenic, India, machine learning, geospatial modeling, random forest

## Abstract

Groundwater is a critical resource in India for the supply of drinking water and for irrigation. Its usage is limited not only by its quantity but also by its quality. Among the most important contaminants of groundwater in India is arsenic, which naturally accumulates in some aquifers. In this study we create a random forest model with over 145,000 arsenic concentration measurements and over two dozen predictor variables of surface environmental parameters to produce hazard and exposure maps of the areas and populations potentially exposed to high arsenic concentrations (>10 µg/L) in groundwater. Statistical relationships found between the predictor variables and arsenic measurements are broadly consistent with major geochemical processes known to mobilize arsenic in aquifers. In addition to known high arsenic areas, such as along the Ganges and Brahmaputra rivers, we have identified several other areas around the country that have hitherto not been identified as potential arsenic hotspots. Based on recent reported rates of household groundwater use for rural and urban areas, we estimate that between about 18–30 million people in India are currently at risk of high exposure to arsenic through their drinking water supply. The hazard models here can be used to inform prioritization of groundwater quality testing and environmental public health tracking programs.

## 1. Introduction

Around the world, but particularly so in India, there is an ever-increasing dependence on groundwater for drinking water supplies and irrigation [1,2]. This is related, in part, to population and economic growth as well as to climate change.

Groundwater is generally much less susceptible to biological and other sources of anthropogenic contamination than is surface water. Its longer residence time and exposure to varying geochemical environments in an aquifer, however, can subject groundwater to the accumulation of various chemical elements in sufficiently high concentrations to pose a health risk to those using it for drinking or cooking [3]. Examples of such naturally occurring (geogenic) contaminants include arsenic, fluoride, manganese, and uranium. Of these, arsenic is one of the most serious contaminants, both in terms of toxicity and ubiquity, which is due to its widespread presence in trace amounts in minerals found in all types of rocks and sediments [3,4]. Many of the large-scale occurrences of geogenic arsenic contamination of groundwater are found in Asia and are due to the release of arsenic found in recently deposited sediments that are exposed to geochemical conditions that are either predominantly reducing (e.g., Ganges-Brahmaputra and Mekong deltas [5]) or oxidizing (e.g., Indus plain [6]). The dominant mobilization mechanisms involve microbially mediated reductive dissolution of host Fe(III) oxyhydroxide minerals and/or reduction of arsenic [7] in reducing environments, while in general intra-aquifer concentrations may be strongly modified by pH- and competitive anion-dependent reversible sorption processes [8]. Other common but less widespread sources or mechanisms of arsenic release into aquifers include the oxidation of sulfide minerals and geothermal activity [9,10].

Long-term exposure to arsenic can lead to various skin diseases, cancers, and cardiovascular diseases [3]. The most common intake pathways include drinking arsenic-contaminated groundwater or consuming high inorganic arsenic crops, particularly rice and those grown in high arsenic soils and/or irrigated with arsenic-contaminated water [11]. Although at least some of the arsenic found in food is present in a less toxic organic form, the arsenic present in groundwater predominantly occurs as one of the more toxic inorganic species, that is, arsenate or arsenite [3]. For this reason, the World Health Organization (WHO) recommends keeping arsenic concentrations in drinking water as low as possible. Although it has a guideline concentration of 10 µg/L, this is only provisional due in part to the difficulties of removing arsenic from water [12]. Likewise, India has set the concentration of 10 µg/L as a requirement (“acceptable limit”), whereas 50 µg/L is kept as a permissible limit in the absence of alternate sources [13].

Aside from some of the well-known arsenic-contaminated areas of India, such as along the Ganges and Brahmaputra rivers in parts of Assam, Bihar, Uttar Pradesh, and West Bengal, groundwater arsenic is not comprehensively tested throughout the country. The complete picture of arsenic contamination in India may therefore not be fully understood. There may also exist in the country areas of arsenic groundwater contamination that have not yet been identified. In order to help determine where high concentrations of arsenic in groundwater may exist in India, we have employed machine learning with a comprehensive dataset of arsenic concentrations and various environmental parameters to produce a model of arsenic-contaminated groundwater for the whole of India. This should indicate where in the country high groundwater arsenic concentrations are likely to be found where no arsenic measurements currently exist. Such an approach has been previously been carried out for the states of Gujarat [14] and Uttar Pradesh [15], as well as the entire world [16], but never solely for all of the country of India or with the substantially larger dataset of arsenic concentrations as in this study. All other factors being equal, a modeling study conducted on a smaller area allows the model to focus on, and better characterize, the arsenic occurrences in that area and thereby produce a more accurate model.

## 2. Materials and Methods 

### 2.1. Arsenic Concentration Measurements

A total of 145,099 geographically distinct arsenic concentration measurements in groundwater were assembled from a multitude of sources including from a systematic compilation of published sources (Table 1). Although the focus of this study is on India, data from adjoining countries, where available, were incorporated to help characterize the occurrence of arsenic in border areas. As such, data acquisition was concentrated on India, which contributed 91% of the data, whereas available datasets from the neighboring countries of Bangladesh (3%), Nepal (5%), and Pakistan (1%) were also included. Reported concentrations were mainly determined by ICP-MS or AA, although some field kit test measurements assured by cross-calibration with laboratory measurements were also included, particularly for areas with otherwise limited data. However, the model sensitivity to the precision of the arsenic measurements in the dataset is lessened by the fact that we convert them to binary format before modeling (see below).

**Figure 1:**
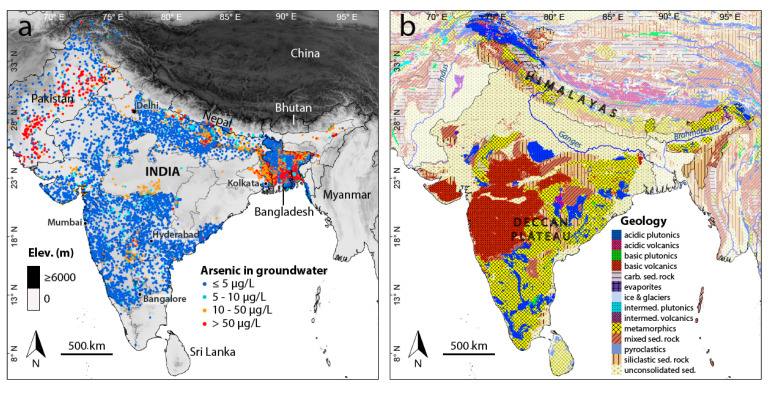
Groundwater arsenic data points and simplified geology of the Indian subcontinent. (**a**) Spatially averaged arsenic data points used in modeling along with topography in India and neighboring countries. (**b**) Lithology of the Indian subcontinent.

As many data are heavily concentrated in a few areas in West Bengal and to a lesser degree Bihar and the Terai (Nepal), an effort was made to reduce the disproportionately high frequency of data coming from these areas and thereby temper their influence on the model. To this end, the concentration measurements were averaged to generate individual data points corresponding to the 1-km × 1-km resolution of the predictor variables where more than one original data point was located within a 1-km × 1-km pixel. This considerably reduced the size of the dataset to 23,799 data points (Figure 1), with the breakdown by country being: India (74%), Bangladesh (15%), Nepal (8%), and Pakistan (3%). The resulting cumulative distribution of arsenic concentrations is shown in Figure S1, with 42% of the concentrations exceeding 10 µg/L. As explained below, a binary target variable was modeled, for which the arsenic concentrations were first recoded to either 0 or 1 according to them being either less than or equal to 10 µg/L or greater than 10 µg/L.

### 2.2. Predictor Variables

Although the target of the modeling is the concentration of arsenic located at some depth from the surface, only parameters determined at the surface are available in a spatially continuous sense across all of India. This is due to the generally high cost and/or difficulty of obtaining relevant subsurface data (e.g., geophysical measurements, drill logs) across the entire country.

In total, 26 different spatially continuous parameters were used as predictor variables in modeling (Table 2). These variables were selected based on their known or perceived function as proxies for the accumulation of arsenic in groundwater [16]. Most of the variables are related to climate or the surface geology, which includes metamorphic and sedimentary rocks in the Himalayas in the north, volcanic and metamorphic units in Deccan plateau in the south, and extensive unconsolidated sediments along the Ganges and Brahmaputra rivers in between (Figure 1b). In addition, included are many soil parameters, which are influenced by both climate and geology, as well as land cover, topography, and water table depth. All but two of the variables are available as 1-km x 1-km rasters (30 arc-second resolution). The two exceptions are the categorical variables of land cover and lithology, both of which are provided as polygon files that were then converted to the same 30 arc-second resolution (1 km at the equator). In preparation for modeling, the geographical coordinates of the 23,799 arsenic data points were used to retrieve the corresponding values of these predictor variables and added to a modeling table.

### 2.3. Prediction Modeling

The arsenic concentration dataset and predictor variables described above were used to create a statistical prediction model of the occurrence of arsenic in groundwater exceeding the WHO and national India guideline concentration of arsenic in drinking water of 10 µg/L. A binary rather than a continuous response variable was chosen due to the anticipated application of the resulting prediction model being to address progressing towards fuller compliance with the 10 µg/L regulatory standard. Furthermore, as we were restricted by necessity to using surface parameters to predict arsenic concentrations at depth, modeling a binary target variable circumvents some of the associated uncertainty and as a consequence should improve the model’s effectiveness.

Although other statistical learning approaches, such as logistic regression and support vector machines, were initially attempted, the random forest method [59] was ultimately adopted due its superior prediction performance in initial tests and was implemented using the R programming language [60]. Random forests are ensembles of decision trees that are grown with elements of introduced randomness. The data used to grow an individual tree are randomly selected by sampling with replacement from the full (training) dataset, which results in about 2/3 of the data rows being utilized, some of which multiple times. Furthermore, the number of predictor variables made available at each branch is restricted and the variables are randomly chosen. Because not all variables are considered simultaneously, any possible effects of multicollinearity among predictors can generally be disregarded in a random forest [61].

The typical number of variables made available at each branch of the trees grown in a random forest is the square root of the number of predictors, which in this case would have been five (taking the square root of 26). This parameter was tuned in order to find the optimal value to use with our dataset by trying values between 1 and 26 (the total number of variables) and comparing the results. This showed that making 10 variables available at each branch produces the most accurate model as measured against the out-of-bag (OOB) data that are randomly sorted out of each tree grown.

For the actual model, the full dataset of 23,799 data points was randomly split into training (80%) and testing (20%) datasets. This was done by stratified sampling so as to maintain the same balance between low and high cases (0 or 1) of the binary target variable, for which 42% of values were greater than 10 µg/L. The training dataset was used to develop the model (encompassing 10,001 trees), which was then cross validated with the testing dataset to determine its accuracy in predicting low (≤10 µg/L) and high (>10 µg/L) arsenic concentrations on new data. The model was then applied to the 26 spatially continuous predictor variables to create a map of the probability of the occurrence of the concentration of arsenic in groundwater exceeding 10 µg/L for all of India.

### 2.4. Importance of Predictor Variables

The effect of the predictor variables on the random forest model was evaluated directly through two statistics: (i) decrease in accuracy and (ii) decrease in Gini node impurity. In calculating both of these, the values of each predictor variable were randomly shuffled in turn and the resulting decrease in accuracy and decrease in Gini node impurity (how well the target variable is split at a branch) were measured on the OOB data of each tree and averaged. A higher (positive) value indicates a greater relative importance of a variable, whereas a negative value (corresponding to greater accuracy or node purity when the values are reassigned) shows that a variable does not benefit a model and should be removed.

As the measures of random forest variable importance described above do not indicate how a predictor variable relates to the target variable, e.g., with a positive or negative trend, Pearson correlations between each (continuous) predictor variable and the proportion of arsenic measurements exceeding 10 µg/L were calculated. This was done by first ordering the values of each predictor and placing them into 16 bins, each containing the same number of values. The proportion of arsenic measurements in each bin greater than 10 µg/L was then calculated. The number of bins was determined using Sturges’ formula (1 + log2 n) [62]. The correlation was calculated between the average value of the predictor in each bin and the proportion of high arsenic concentrations. 

### 2.5. Estimating Potentially Affected Population

The prediction map generated from the random forest model was subsequently used to estimate the population potentially exposed to high concentrations of arsenic in drinking water. The first step of this procedure was to determine the areas at high risk of having arsenic concentrations greater than 10 µg/L. This was done using two approaches that have been described in more detail by Podgorski and Berg [16]. The sensitivity and specificity, that is, the correct classification of high and low values, respectively, were plotted for 100 probabilities between 0 and 1. Where both curves intersect indicates the probability cutoff at which the model classifies low and high values equally well. The second approach that was used follows a similar procedure that instead finds the intersection of the positive predictive value (PPV) and negative predictive value (NPV), which are defined as rate of correct positive and negative predictions, respectively. These analyses for determining how to interpret the model were conducted with all available data (training and testing datasets). Each probability threshold found with these two approaches was subsequently used to identify high hazard areas on the probability map. The two sets of high hazard areas were then used for further calculations to produce a range of values of populations at risk.

The populations living in the high hazard areas determined according to the above procedure were then multiplied by the modeled probabilities. The population figures were taken from a population model for 2020 based on existing patterns of development [63]. The at-risk population was further refined by differentiating between rural and urban areas [55] and multiplying by recent (2016) estimated nationwide usage rates of untreated groundwater in rural (0.637) and urban (0.238) areas, respectively [64].

## 3. Results and Discussion

### 3.1. Arsenic Prediction Model

The cross-validation results of the final random forest model as applied to the test dataset are shown in Table 3. The area under the ROC (receiver operator characteristic) curve (AUC) is 0.86, which in general can range between 0.5 (random model) and 1 (perfect model) and represents how well a binary model can predict both low and high values as assessed over numerous probability cutoff values [65]. The AUC of this model is generally a better result than that of similar regional or country-scale groundwater quality studies (where reported), for example, 0.84 for fluoride in India [66], 0.71–0.83 for arsenic in Gujarat [14], 0.82 for arsenic in the USA [67], 0.80 for arsenic in Pakistan [6], or 0.74 for arsenic in Uttar Pradesh [15]. The overall accuracy of the model as applied to the test dataset of 0.79 is significantly higher than the no information rate of 0.58 (*p* value < 2.2 × 10^−16^) and is comparable to the average accuracy with OOB samples of 0.77. The no information rate refers to the accuracy that would be achieved without a model and is simply the proportion of the more frequent class of the dataset, i.e., 58% of the arsenic measurement points are equal to or less than 10 µg/L. Similarly, the Cohen’s kappa statistic [68] (0.5606) is an indicator of accuracy beyond what could be expected by chance and varies from 0 (no agreement) to 1 (perfect agreement).

Despite the spatial averaging of data to 1-km pixels, there still remains a much higher density of data points in a few regions, particularly in West Bengal where nearly 50% of the averaged data points are located. In order to test if such an imbalance considerably biases the model by allocating excessive weight to the conditions found in a particular region, the data from West Bengal were randomly split into 10 subsets that were each modeled separately with the rest of the dataset. The results of the 10 different models were averaged and compared with the single model using the full dataset (containing all West Bengal data). As the model performance was essentially identical, e.g., AUC of 0.86 and balanced accuracy of 0.78, the simpler approach of using a single model was retained and that of splitting the West Bengal data was not further pursued.

Early attempts to separately model areas corresponding to reducing environments, arid-oxidizing environments, and sulfide oxidation arsenic mobilization processes did not result in considerably different predictions or accuracy and were therefore abandoned in favor of a single model for India. It appears that the single model is able to effectively account for different geochemical environments due to utilizing the same parameters of climate, geology, and soil pH that were used to define the different environments. For example, in the ten thousand trees that make up the random forest, splits would have been made on significant differences in these parameters, which is similar in principle to having done so manually.

**Figure 2:**
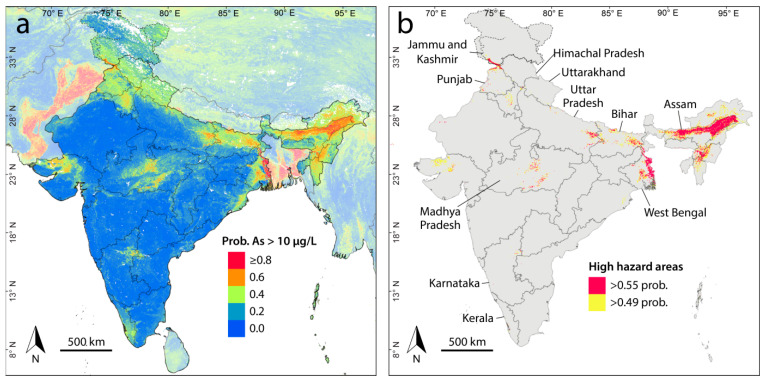
Arsenic hazard maps. (**a**) Probability of arsenic concentration in groundwater exceeding 10 µg/L. (**b**) High hazard areas in India based on probability cutoffs of 0.49 and 0.55.

The arsenic prediction map generated from the final random forest model is displayed in Figure 2a. As most of the data used in the model is from India with the data from neighboring countries having been incorporated merely to help characterize border areas, the analysis that follows will focus only on India. Nonetheless, the arsenic prediction map including the rest of South Asia is presented in Figure S2, which, as seen with model continuity across international borders, also serves as a reminder that political boundaries do not necessarily coincide with those of natural systems.

The prediction model (Figure 2a) captures known arsenic-prone areas in the alluvial sediments along the Ganges and Brahmaputra plains [27] and in Gujarat [14] and Punjab [43]. It also identifies less well-known or previously undocumented areas such as Haryana, Jammu and Kashmir, and central Madhya Pradesh. Elevated arsenic hazard also appears in Himachal Pradesh, Kerala, and Uttarakhand, from where no arsenic concentration data were available to us. Consistent with the findings of Sovann and Polya [69] for Cambodia, this confirms that small alluvial systems, in addition to the larger systems, such as those of the Ganges, Brahmaputra, and Mekong, may also host elevated groundwater arsenic concentrations. This highlights some of the advantages and uses of a prediction model based on machine learning rather than spatial interpolation. That is, predictions can be made in areas removed from where concentration measurements exist by relying on statistical relationships between measurements in other areas and predictor variables that cover the entire model domain. The model can then be used to prioritize areas for testing where there is a dearth of data. The results of new water quality testing can then in turn be fed back into the model to further improve it. 

### 3.2. Influence of Predictor Variables

**Figure 3:**
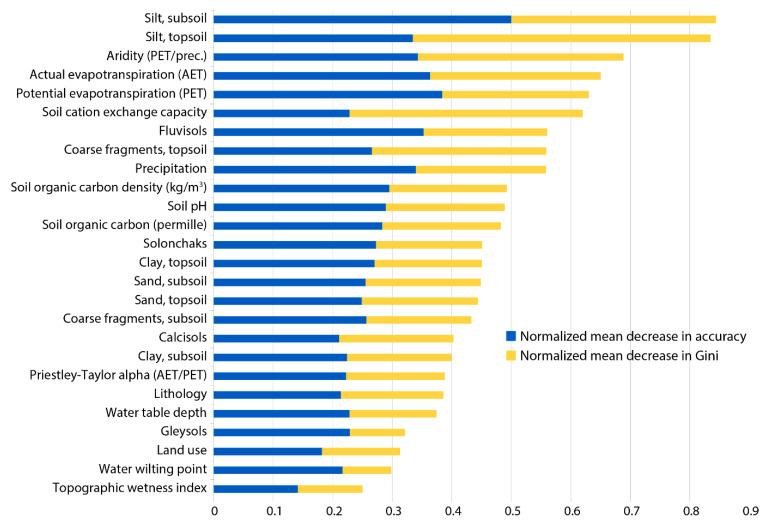
Normalized variable importance in terms of mean decrease in accuracy and mean decrease in Gini as calculated on the test dataset. Both decrease in accuracy and decrease in Gini were normalized by their respective greatest values (see Table S1).

The importance of the predictor variables in the random forest model was assessed as the mean decrease in accuracy and mean decrease in Gini impurity. Each of these was normalized by the maximum value calculated among the predictor variables, and both are displayed together in Figure 3. This shows both silt variables (subsoil and topsoil) placing markedly above the others in importance, followed by aridity and both actual and potential evapotranspiration. The least important variables relative to the others are topographic wetness index, water wilting point, gleysols, and land use. Nevertheless, none of the predictors has a negative importance value, which suggests that they are all beneficial to the model.

**Figure 4:**
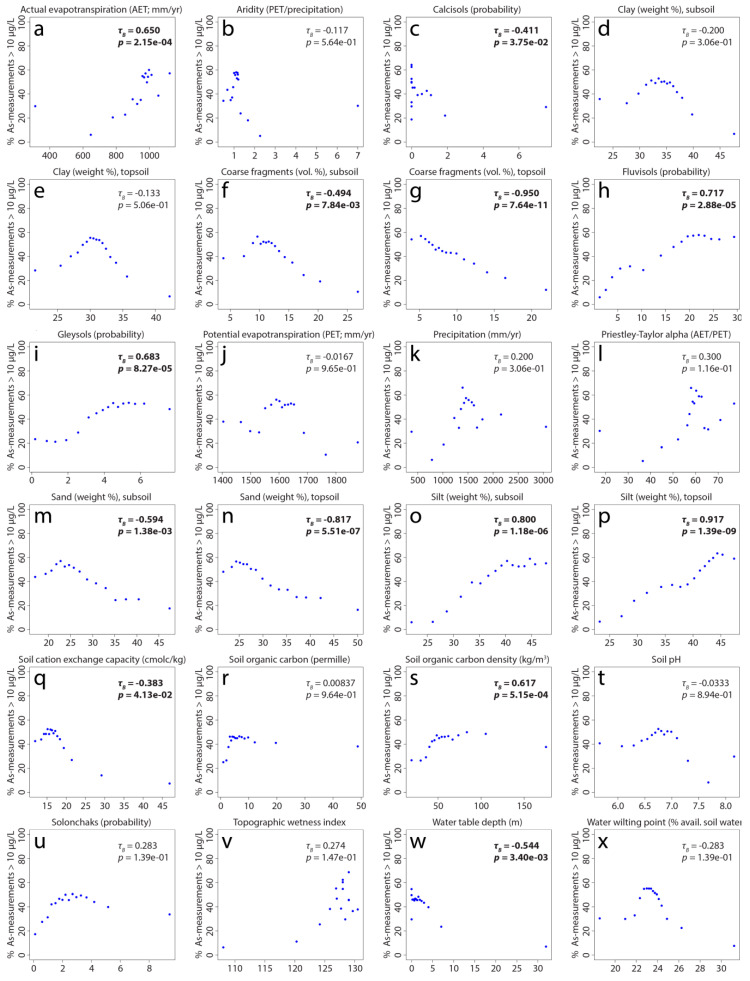
Correlations of predictor variables (**a**–**x**) with percentages of arsenic data points exceeding 10 µg/L in 16 equally sized bins. Kendall correlations (τ_B_) with a statistically significant *p* value (95% confidence level) are shown in bold.

In order to better understand how the predictor variables may relate to high arsenic concentrations (>10 µg/L), the proportion of high arsenic measurements were plotted against the averages of binned predictor values, for which rank-order correlations (Kendall Tau-b) were also calculated (Figure 4). The strongest rank-order correlations were found with topsoil coarse fragments (−0.95), silt in both the topsoil (0.92) and subsoil (0.80), topsoil sand (−0.82), fluvisols (0.72), gleysols (0.68), and actual evapotranspiration (0.65). Well-defined peaks are also observed with subsoil and topsoil clay (Figure 4d,e), subsoil coarse fragments (Figure 4f), subsoil and topsoil sand (Figure 4m,n), soil cation exchange capacity (Figure 4q), soil pH (Figure 4t), and water wilting point (Figure 4x), which highlights why it can be important to use a nonlinear classifier for modeling, such as random forest, to capture such relationships. 

The importance of gleysols and their positive correlation with high arsenic concentrations can be linked to chemically reducing conditions, which are conducive to arsenic release [7,9], brought about by the poor drainage associated with this soil type. The negative relationship between soil cation exchange capacity and groundwater arsenic is at first glance counterintuitive given that higher CEC may give rise to higher pHs at which anionic, deprotonated As(III) or As(V) species will be more likely to desorb. Accordingly, this relationship might instead reflect the typically higher clay/silt contents of high CEC soils. The inverse correlation with water table depth is likely related to the occurrence of higher arsenic concentrations in the shallower Holocene layers of alluvial sedimentary systems [70] and that tend to contain higher concentrations of labile reactive organics associated with arsenic mobilization [71,72,73]. Furthermore, the strong dependence of the model on the silt content of soil, including a positive relationship with high arsenic concentrations, points toward the occurrence of high arsenic concentrations beneath floodplains, as do similar relationships with fluvisols.

Many of the observations made above are compatible with each other and are consistent with the reductive dissolution of arsenic in known high arsenic-hazard areas of Figure 2a found within unconsolidated sediments (Figure 1b), particularly along the Ganges and Brahmaputra rivers. Notable exceptions to this are the arsenic areas in central Madhya Pradesh (Figure 2a), which occur primarily within mafic volcanic rocks as well as the high hazard areas located in acidic plutonic rocks (e.g., granite) in Kerala and northeast Karnataka. The last example may be due to the oxidation of arsenic-bearing sulfide minerals, which is likely responsible for high arsenic concentrations in areas of historical gold mining activity in Karnataka [74].

### 3.3. Populations at Risk

**Figure 5:**
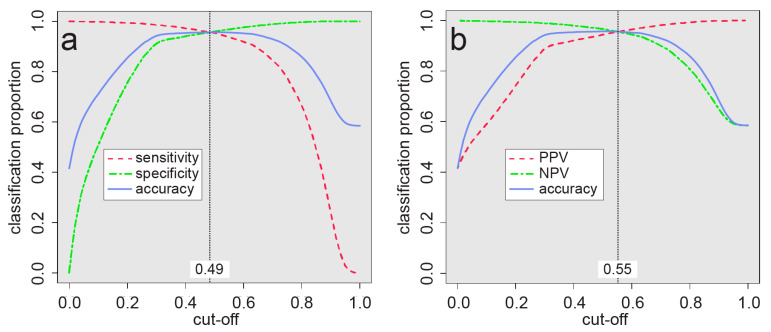
Analyses of model performance using full modeling dataset. (**a**) Sensitivity and specificity were found to be equivalent at a probability cutoff of 0.49 with a corresponding accuracy of 96%. (**b**) Positive predictive value (PPV) and negative predictive value (NPV) were found to be equivalent at a probability cutoff of 0.55 also with a corresponding accuracy of 96%.

The estimated arsenic-risk areas based on the probability cutoffs of 0.49 and 0.55 (see Figure 5) are shown in Figure 2b, in which most of the risk areas are seen to be concentrated along the Brahmaputra river (Assam) and the lower half of the Ganges river (Uttar Pradesh, Bihar, and West Bengal). Other notable risk areas are found in Jammu and Kashmir, Punjab, Gujarat, Madhya Pradesh, and most of the states neighboring Assam. The proportion of land area and populations that are potentially affected are broken down by state/territory in Table 4. Because only grid cells with a probability exceeding 0.49 or 0.55 were considered, underestimation may occur where there is localized arsenic contamination. In total, we estimate that between about 18 and 30 million people in India may be currently consuming arsenic in groundwater at concentrations exceeding 10 µg/L. This population estimate is substantially lower than that estimated (~41 million) for the five most impacted Indian states by Chakraborti et al. [75] and is consistent with the estimate of 31 million derivable from Ravenscroft et al. [9]. It is difficult to state possible reasons for these differences as the methods used to calculate these other estimates are not clear. Our range of potentially affected population is also toward the lower end of the range of 18–90 million estimated for India as part of a global groundwater arsenic prediction model [16], which highlights how a separate study, such as this one, concentrated on a single area can lead to more precise results.

## 4. Conclusions

The purpose of the hazard models produced here is to offer an overview of where high concentrations of geogenic arsenic are likely to be found in groundwater across the whole of India, with some insight into the physical processes at work, as well as to assess the size of the populations potentially affected. Particularly since arsenic is often not routinely analyzed in many areas without a known problem, the maps should serve as a guide to identifying where additional testing should be conducted as well as in assessing health impacts. In addition to drinking water supplies, the hazard map is relevant to the utilization and siting of wells for the irrigation of crops.

Although this study is based on over 145,000 groundwater samples, targeted testing is still required to determine if a specific well is highly contaminated with arsenic or not. This is due, at least in part, to the heterogeneous nature of aquifers that can lead to great variability of groundwater arsenic concentrations over short distances. Further, additional groundwater arsenic concentration data would help improve the model, particularly in areas (e.g, in parts of Maydha Pradesh) in which anthropogenic processes may have had a material influence on groundwater arsenic concentrations and thereby the rendered model.

Despite the hazard model presented here being very effective in predicting high groundwater arsenic concentrations, it is unable to account for the depth dependency of arsenic in an aquifer, which, for example, can vary according to sediment age and/or redox conditions as well as 3-D heterogeneous permeability structures [76]. As such, it can be assumed that the predictions become less accurate with greater depth, as was demonstrated in a recent global study [16]. Although ~3/4 of the modeled data points used here have an associated well depth, essentially all of these data were confined to just a few areas in West Bengal and Bihar. Given the size of India and its variations in climate and geology, it was not feasible to generalize depth relationships based on data from just a small part of the country. However, incorporating depth as a predictor variable could be effective in a smaller-scale study of a single region.

Another dimension that could enhance the modeling is that of time. For example, accounting for fluctuations in arsenic concentrations relative to the monsoon season could possibly improve the accuracy of the model, particularly in areas with extensive hyporheic zones undergoing surface water–groundwater exchange and particularly including those proximal to rivers with large differences in post- and pre-monsoonal stages. Any long-term secular trends in arsenic concentration related to aquifer exploitation [77,78,79,80], aquifer depletion, or climate change could also produce useful results. This was not possible in this study due to each of the arsenic measurement points representing spatially distinct locations that also generally lacked information on the timing of sampling.

The population estimates rely on a single country-wide set of groundwater-use rates for rural and urban areas. Knowing this rate at a finer scale (e.g., state, division, and district) could be expected to lead to more accurate results. In order get a better grasp of who is physically impacted by high groundwater arsenic concentrations, exposure studies and environmental public health tracking [81] leveraging the arsenic hazard map produced here would be very helpful.

## Figures and Tables

**Table 1:** Summary of groundwater arsenic concentration data used in the model. Existing arsenic measurements taken from over 30 sources, mainly from India but also from some neighboring South Asian countries. Summaries are given for before and after spatial averaging.

Country [Data Sources]	Number of Data Points, before Spatial Averaging (% of Total)	Mean (±Standard Deviation) As Concentration, before Spatial Averaging	Number of Data Points, after Spatial Averaging (% of Total)	Mean (±Standard Deviation) As Concentration, after Spatial Averaging
India [17,18,19,20,21,22,23,24,25,26,27,28,29,30,31,32,33,34,35,36,37,38,39,40,41,42,43,44,45,46,47]	132,028 (91%)	53 ± 451 µg/L	17,528 (74%)	33 ± 162 µg/L
Bangladesh [42,48]	4215 (3%)	62 ± 139 µg/L	3674 (15%)	56 ± 120 µg/L
Nepal [49]	7575 (5%)	15 ± 62 µg/L	1846 (8%)	16 ± 66 µg/L
Pakistan [6,50]	1279 (1%)	103 ± 123 µg/L	760 (3%)	71 ± 98 µg/L
Total	145,097	52 ± 438 µg/L	23,808	37 ± 150 µg/L

**Table 2:** Predictor variables and descriptions. The 26 parameters used as predictor variables in modeling are grouped into the categories ‘Climate’, ‘Soil’ and ‘Other’. Their resolution is one square km at the equator.

Variable	Description
**Climate**	
Actual evapotranspiration (AET) [51]	Average rate of actual evapotranspiration (mm/yr)
Aridity	PET/Precipitation
Potential evapotranspiration (PET) [52]	Average rate of potential evapotranspiration (mm/yr)
Precipitation [53]	Average rate of precipitation (mm/yr)
Priestley-Taylor alpha coefficient [51]	AET/PET
**Soil**	
Calcisols [54]	Probability of the occurrence of calcisols
Clay, subsoil [54]	Weight % of clay particles (<0.0002 mm) at 2 m depth
Clay, topsoil [54]	Weight % of clay particles (<0.0002 mm) at 0 m depth
Coarse fragments, subsoil [54]	Volumetric % of coarse fragments (>2 mm) at 2 m depth
Coarse fragments, topsoil [54]	Volumetric % of coarse fragments (>2 mm) at 0 m depth
Fluvisols [54]	Probability of the occurrence of fluvisols
Gleysols [54]	Probability of the occurrence of gleysols
Sand, subsoil [54]	Weight % of sand particles (0.05–2 mm) at 2 m depth
Sand, topsoil [54]	Weight % of sand particles (0.05–2 mm) at 0 m depth
Silt, subsoil [54]	Weight % of silt particles (0.0002–0.05 mm) at 2 m depth
Silt, topsoil [54]	Weight % of silt particles (0.0002–0.05 mm) at 0 m depth
Soil cation exchange capacity [54]	Cation exchange capacity (cmolc/kg) at 2 m depth
Soil organic carbon [54]	Soil organic carbon (permille) at 2 m depth
Soil organic carbon density [54]	Soil organic carbon density (kg/m^3^) at 2 m depth
Soil pH [54]	Soil pH measured in water at 2 m depth
Solonchaks [54]	Probability of the occurrence of solonchaks
Water wilting point [54]	Vol. % of available soil water until wilting point at 2 m depth
**Other**	
Land cover [55]	17 different land cover categories according to the International Geosphere-Biosphere Programme (IGBP)
Lithology [56]	15 different categories of lithology
Topographic wetness index [57]	Combination of upslope contributing area and slope
Water table depth [58]	Mean water table depth (m)

**Table 3:** Confusion matrix and other statistics resulting from the analysis of the final random forest model with the test dataset at a probability cutoff of 0.5.

	**Reference**	
**Prediction**	**0**	**1**
**0**	2223	462
**1**	561	1514
**Statistic**	**Value**	
Accuracy (Acc)	0.7851	
No information rate (NIR)	0.5849	
*p* value (Acc > NIR)	<2.2 × 10^−16^	
Cohen’s kappa	0.5606	
Sensitivity	0.7662	
Specificity	0.7985	
Positive predictive value	0.7296	
Negative predictive value	0.8279	
Prevalence	0.4151	
Balanced accuracy	0.7823	

**Table 4:** Area and population potentially exposed to arsenic concentrations greater than 10 µg/L by state/territory. Based on probabilities in Figure 2a exceeding 0.49 and 0.55 along with the rates [64] of household groundwater use in rural and urban areas. See text for limitations.

State/Territory	Percentage of Land Area Exposed	Population Exposed
Andaman and Nicobar	0.4–2.9%	300–2700
Andhra Pradesh	<0.1%	2700–6600
Arunachal Pradesh	4.3–21.6%	69,800–157,700
Assam	42.3–59.7%	6,536,000–8,771,100
Bihar	3.0–12.0%	1,226,800–4,636,500
Chandigarh	n/a	n/a
Chhattisgarh	<0.1%	700–1100
Dadra and Nagar Haveli and Daman and Diu	n/a	n/a
Delhi	n/a	n/a
Goa	n/a	n/a
Gujarat	0.3–4.0%	19,300–97,300
Haryana	0.4–5.0%	39,200–447,200
Himachal Pradesh	0.4–0.9%	36,800–76,900
Jammu and Kashmir	0.7–1.1%	337,800–470,800
Jharkhand	0.2–0.6%	103,600–231,400
Karnataka	0.1–0.5%	29,400–93,900
Kerala	<0.4%	10,400–77,300
Madhya Pradesh	0.7–2.1%	201,200–552,100
Maharashtra	<0.1%	300–1700
Manipur	8.6–22.7%	46,500–121,900
Meghalaya	0.2–1.5%	3300–13,800
Mizoram	4.5–18.0%	23,500–82,400
Nagaland	5.9–21.5%	54,300–188,600
Odisha	<0.4%	1300–194,600
Puducherry	n/a	n/a
Punjab	2.3–6.8%	299,100–788,500
Rajasthan	<0.1%	2300–10,800
Sikkim	n/a	n/a
Tamil Nadu	<0.1%	200–200
Telangana	<0.2%	4100–12,900
Tripura	0.1–1.4%	800–10,000
Uttar Pradesh	1.0–2.4%	1,222,800–2,458,500
Uttarakhand	<0.7%	900–42,300
West Bengal	12.9–20.5%	7,432,200–10,144,700
Total	2.0–4.2%	17,710,000–29,690,000

## Supplementary Materials

The following are available online at https://www.mdpi.com/1660-4601/17/19/7119/s1, Figure S1: Cumulative frequency of groundwater arsenic concentrations, Figure S2: Prediction model of arsenic in groundwater for South Asia, Table S1: Importance of predictor variables.
