# Supplementary material for: Groundwater Arsenic Distribution in India by Machine Learning Geospatial Modeling
Source: Int J Environ Res Public Health. 2020 Sep 28;17(19):7119. doi: 10.3390/ijerph17197119 (PMC7579008; doi:10.3390/ijerph17197119)
Supplement: Supplementary file 1 [file ijerph-17-07119-s001.pdf]

International Journal of
Environmental Research and Public Health

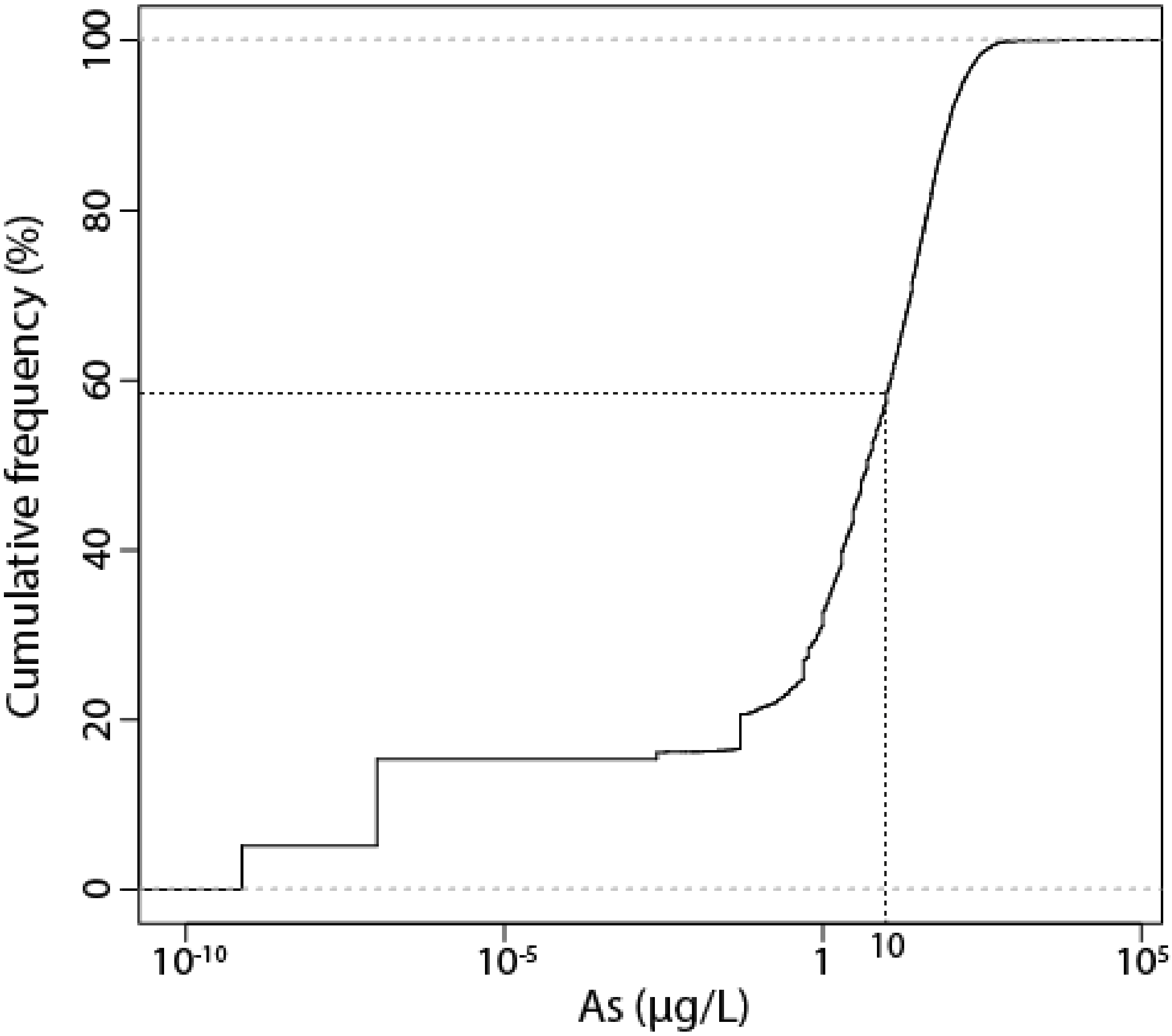

Figure S1. Cumulative frequency of groundwater arsenic concentrations of the averaged data points used in modeling (*n* = 23,799). About 42% of the arsenic data are greater than 10 µg/L.

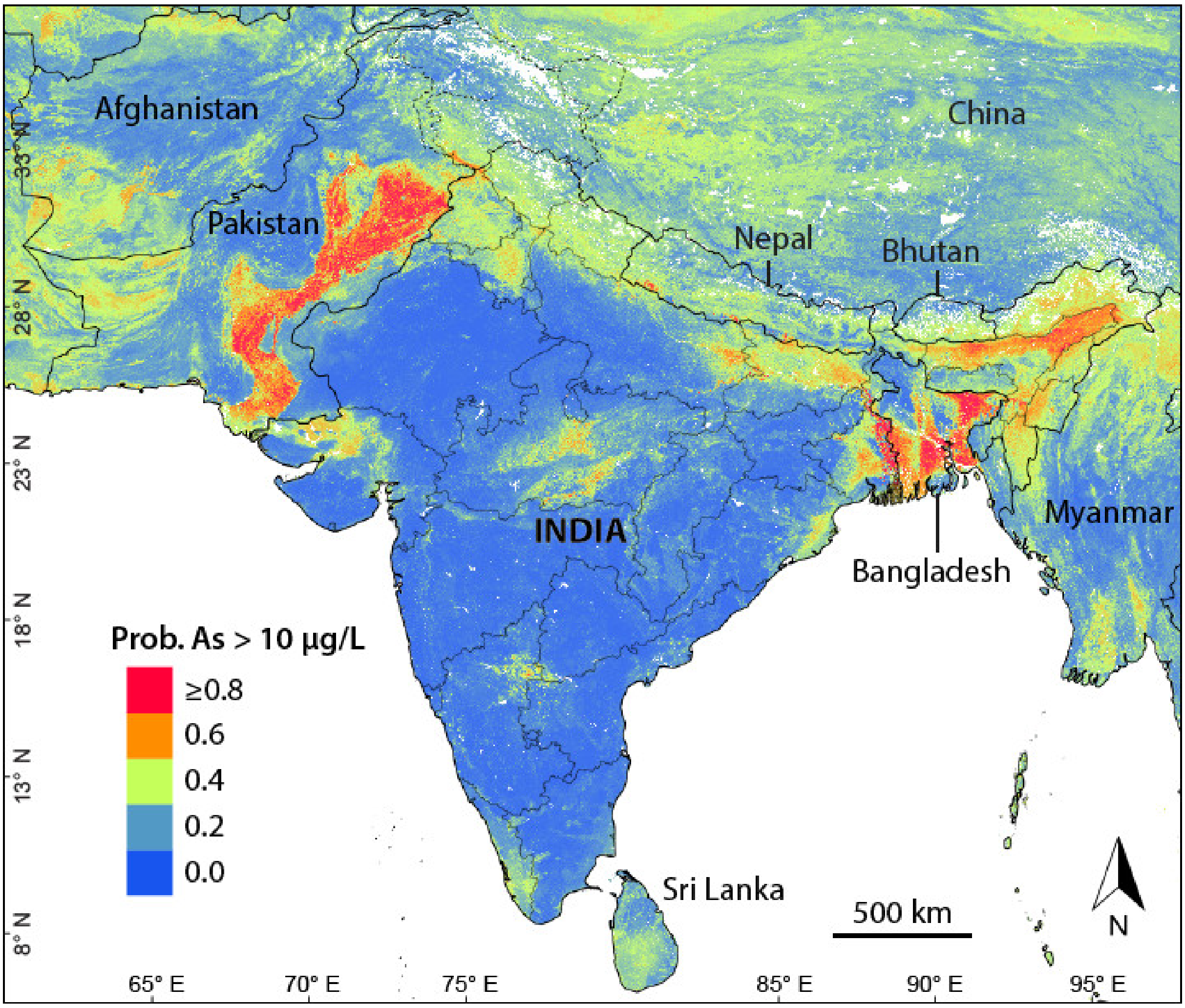

Figure S2. Prediction model of arsenic in groundwater exceeding 10 µg/L as created for the South Asian region using the data points depicted in Figure 1a.

Table S1. Importance of predictor variables in the random forest model in terms of mean decrease in accuracy and mean decrease in Gini as well as correlation (*r*) of variables with percentage of measurements greater than 10 μg/L and associated significance level (*p*).

Variable	Mean Decrease in Accuracy	Mean Decrease in Gini	*r*	*p*
Actual evapotranspiration (AET)	254.334	537.130	0.636	8.04E-03
Aridity (PET/PREC)	269.428	445.505	−0.323	2.23E-01
Calcisols	169.521	143.980	−0.360	1.71E-01
Clay, subsoil	158.388	268.086	−0.468	6.75E-02
Clay, topsoil	209.855	309.710	−0.378	1.49E-01
Coarse fragments, subsoil	184.352	303.205	−0.773	4.50E-04
Coarse fragments, topsoil	188.981	301.466	−0.986	2.27E-12
Fluvisols	169.396	608.247	0.938	7.63E-08
Gleysols	190.137	274.182	0.881	6.76E-06
Land use	160.532	126.917	n/a	n/a
Lithology	134.900	204.250	n/a	n/a
Potential evapotranspiration (PET)	370.450	534.824	−0.327	2.16E-01
Precipitation	284.738	382.778	0.262	3.26E-01
Priestley-Taylor alpha (AET/PET)	164.911	258.049	0.522	3.79E-02
Sand, subsoil	200.388	280.023	−0.880	6.84E-06
Sand, topsoil	202.082	277.140	−0.940	6.68E-08
Silt, subsoil	248.087	777.329	0.945	3.63E-08
Silt, topsoil	197.022	455.448	0.972	3.23E-10
Soil cation exchange capacity	156.104	299.381	−0.879	7.36E-06
Soil organic carbon (permille)	218.742	306.5007	0.00243	9.93E-01
Soil organic carbon density (kg/m^3)	251.539	340.550	0.334	2.05E-01
Soil pH	261.175	323.234	−0.458	7.43E-02
Solonchaks	214.209	310.609	0.173	5.22E-01
Topographic wetness index	105.063	168.496	0.702	2.41E-03

Water table depth	166.113	272.519	−0.851	2.96E-05
Water wilting point	169.060	227.561	−0.441	8.77E-02

© 2020 by the authors. Licensee MDPI, Basel, Switzerland. This article is an open access article distributed under the terms and conditions of the Creative Commons Attribution (CC BY) license (http://creativecommons.org/licenses/by/4.0/).
